# Disparities in women’s cancer-related quality of life by Southern Appalachian residence

**DOI:** 10.1007/s11136-018-1793-8

**Published:** 2018-01-27

**Authors:** Ann L. Coker, Huong T. Luu, Heather M. Bush

**Affiliations:** 1 Endowed Chair, Center for Research on Violence Against Women, University of Kentucky, Lexington, USA; 2 College of Public Health, University of Kentucky, Lexington, USA; 3 Center for Research on Violence Against Women, Lexington, USA; 4 Department of Obstetrics and Gynecology, University of Kentucky, 800 Rose St C-361, Lexington, KY 40536-0293 USA

**Keywords:** Rural, Quality of life, Cancer, Violence, Disparities

## Abstract

**Purpose:**

The purpose was to determine whether Appalachian residence alone or in combination with violence was linked to poorer quality of life (QOL).

**Methods:**

Women recently diagnosed and included in either the Kentucky or North Carolina Cancer Registries were interviewed by phone between 2009 and 2015 (*n* = 3320; mean age = 56.74). Response rates were similar by state (40.1 in Kentucky and 40.9% in North Carolina). Appalachian (*N* = 990) versus non-Appalachian residents (*N* = 2330) were hypothesized to have poorer QOL defined as (a) lower Functional Assessment of Cancer Therapy—General (FACT-G) scores and (b) more symptoms of depression, stress, or comorbid physical conditions. Lifetime intimate partner or sexual violence was first investigated as a moderator then mediator of regional differences. Multiple analyses of covariance (MANCOVA) models were used.

**Results:**

Violence modified the effect of Appalachian residence on poorer QOL outcomes; FACT-G total scores (*p* = .02) were lowest for women living in Appalachia who had additionally experienced violence. Socioeconomic indicators appeared to mediate or explain differences in QOL outcomes by Appalachian residence such that when adjusting for income, education and insurance, Appalachian residence remained associated only with poorer physical QOL outcomes (*p* < .05).

**Conclusions:**

While violence rates did not differ by residence, the combined effect of living in Appalachia and experiencing violence resulted in significantly greater impact on poorer QOL among women recently diagnosed with cancer. Clinical consideration of patients’ residence, socioeconomic status and violence experienced may help identify and mitigate the longer-term impact of these identifiable factors associated with poorer QOL.

## Introduction

Appalachia is recognized by the National Institute on Minority Health and Health Disparities as an under-resourced and medically underserved region due in part to its physical isolation, rurality, and poverty. Southern and rural Appalachia has particularly been characterized by disparities in cancer risk and mortality [1]. Residents in Appalachian counties have the nation’s highest death rate from lung cancer and are more likely to die within 3 to 5 years of their cancer diagnoses than those in both urban Appalachian areas and urban areas [2]. From 1980 to 2014 cancer mortality rates have declined across the USA by 20.1% but not in Appalachian Kentucky, where lung cancer mortality rates *increased* during this period of national decline, and cancer mortality rates were one-third higher than that for urban, non-Appalachian residents [3]. National declines over time in largely preventable cervical cancer incidence [4] have occurred yet rates remain higher in Appalachia [5].

In addition to poverty and physical isolation, Appalachian Kentucky in particular has long been characterized by reduced health care access [6–9] yet the needs for health care are greater in this region. Rates of tobacco use, limited exercise, and poor diet are among America’s highest in Appalachian Kentucky [10, 11] and these adverse health behaviors are well known to be associated with chronic diseases including cancer [12]. Given the isolation, poverty, and limited employment options, migration of younger residents has occurred leaving older residents who are at increased risk of cancer [11]. Despite the increased need for health care professionals, this region has fewer professionals and cancer specialists in particular [9]. Lastly despite evidence that patient navigators are effective in improving cancer diagnostic outcomes particularly in medically underserved areas [13], Anderson et al. [14] found that many cancer care centers in Appalachia did not have patient navigator services. This finding provides additional evidence that those diagnosed with cancer in Appalachian may be at greater risk of poorer cancer outcomes.

Living in Appalachia may also be associated with poorer quality of life (QOL) yet few studies have addressed this potential disparity. The majority of studies to investigate the impact of geography on QOL have examined rural versus urban disparities in QOL following cancer diagnosis, and their findings were somewhat consistent. Only Schultz and Winstead-Fry [15] observed that rural cancer patients evaluated within one to three months after diagnosis had higher mean Functional Assessment of Cancer Therapy-General (FACT-G) scores, indicating *better* QOLL, than those living in urban areas. In contrast, Beck et al. [16] observed that the physical component score (MOS SF-12) and the Functional Performance Inventory were lower, indicating poorer QOL, among rural versus urban cancer survivors. Consistent with findings reported by Beck et al. [16], Reid-Arndt and Cox [17] found that women diagnosed with breast cancer and living in rural regions of the American Midwest had lower overall QOL score (*p* = .03) than did women living in non-rural areas after adjusting for age and education. Turning to psychological QOL, Burris and Andrykowski [18] found that rural Kentucky cancer survivors had poorer mental health functioning, greater symptoms of anxiety and depression, greater distress, and more emotional problems than did their non-rural counterparts. Likewise, Weaver et al. [19] observed that rural cancer survivors were significantly (*p* < .001) more likely to report poorer health status and greater psychological distress than their urban counterparts.

While income contributes to health disparities, few studies have evaluated the effects of income on cancer survivors’ health and QOL. In one such study Short and Mallonee [20] created an income proxy measure using data available from cancer registries including home ownership, sources of unearned income, marital status at diagnosis, and spousal characteristics. This proxy measure was associated with lower FACT-G and SF-12 scores. Given socioeconomic driven disparities in QOL and poorer QOL in Southern and rural Appalachian, where incomes were generally lower, additional research is needed to directly measure household income and evaluate the potential mediational effect of income on rural Appalachian residence and its effect on QOL. An inability to measure and adjust for individual income could confound the effect of Appalachian residence on health outcomes including cancer QOL.

Data for this analysis originated from a larger National Institutes of Health-funded study focusing on life stresses including violence against women and access to cancer care and survivorship. Because cancer survival rates are lower in Appalachia, this grant focused on whether violence might explain poorer cancer outcomes among women living in Appalachian versus non-Appalachian counties. In prior analyses using these data, women who had experienced intimate partner violence (IPV) or sexual violence (SV) had poorer QOL than did women never experiencing these forms of violence [21]. The research questions for the current analysis were (1) ‘do women recently diagnosed with cancer who reside in Appalachian Kentucky or North Carolina have poorer QOL than women living in non-Appalachian counties?’ and (2) ‘does violence modify (or mediate) the association between Appalachian residence and QOL?’.

Because personal interview data was available from women recruited for cancer registries the authors had direct data to measure and adjust models for women’s current and past violence, socioeconomic status including current household income, marital status, private health insurance, and lifetime educational attainment. This research expands research on Appalachian disparities by measuring a range of QOL domains.

Women living in Appalachian counties who were recently diagnosed with an incident, primary, biopsy-confirmed cancer were hypothesized to have lower self-reported QOL defined as (a) lower Functional Assessment of Cancer Therapy-General (FACT-G) total and by domain scores, and (b) poorer mental health (more symptoms of stress or depression) and more comorbid conditions at cancer diagnosis relative to those not living in Appalachian counties. Because prior analyses indicated violence to be strongly correlated with poorer QOL, IPV/SV was explored first investigated as a moderator of the hypothesized effect of Appalachian residence on QOL outcomes to determine whether violence and Appalachian residence had a combined deleterious effect on poorer QOL. The ultimate measure of effect for association between Appalachian residence on QOL outcomes was investigated adjusting for identified confounders.

## Methods

Women ages 18–79 (mean age = 56.74) diagnosed with an incident and primary cancer in the prior 12 months and included in either the Kentucky Cancer Registry (KCR) or the North Carolina Central Cancer Registry (NCCR) were eligible for this study. KCR case recruitment dates ranged from November 2009 to December 2013 and NCCR dates ranged from October 2013 to January 2015. Both KCR and NCCR verified pathology reports. NCCR was recruited to provide sufficient cases living in Appalachia therefore Appalachian North Carolina counties were oversampled. All 28 Appalachian counties of North Carolina (westernmost region) were eligible as were cases living in one of 46 non-Appalachian NC counties adjacent to but east of these 28 Appalachian NC counties. Given differences in the two cancer registries our approach to contacting cases differed somewhat such that KCR directly contacted eligible women by mail and, if no response, by phone to ensure that only those providing consent be contacted by researchers. In contrast North Carolina law allows researchers to directly contact those included in NCCR provided the study was approved and IRB obtained. Thus NCCR staff provided case contact information directly to research staff at the University of Kentucky (UK) for study recruitment. A letter describing the study was sent to all women in both registries. The letter invited women to provide their contact information (name and phone number) on an enclosed card, stamped and addressed to research staff. Women could also indicate that they did not wish further contact on the same card; these women were not contacted. If physicians provided a reason his/her patient should not be contacted (i.e. dementia, death, or too ill to participate) these women were also not contacted. Trained research staff at the UK Survey Research Center (SRC) called participants to explain the study and answer any questions. When participants were reached by phone, the interviewer provided the study introduction, answered questions, and obtained explicit verbal consent before beginning the interview. Phone interviews were attempted within 1 year of cancer diagnosis (average time at completed was 12.93 months, range 2–26). Those completing the interview received a $10.00 incentive. This study was approved by the IRB at the University of Kentucky, protocol number 09-0685-F1V and an NIH Certificate of Confidentiality was granted (MD-09-007).

## Measures

The independent variable, Appalachian or other county of residence was provided by the cancer registry and defined using designation from the Appalachian Regional Commission (County Economic Status in Appalachia, FY 2015. 2014; http://www.arc.gov/research/MapsofAppalachia.asp?MAP_ID=90).

Dependent variables included Functional Assessment of Cancer Therapy-General (FACT-G) total score and by domains, Functional Assessment of Chronic Illness Therapy-Spiritual Well-being (FACIT-SP) scores, self-reported symptoms of stress, and number of comorbid conditions. For all outcomes the time frame for recall was within the month before interview to reflect current health.

Information to characterize self-reported QOL was provided by participants during phone interviews. These included a 27-question FACT-G questionnaire [22] (Cronbach’s *α* = 0.90). FACT-G measured physical (7 items: range 0–21; mean (M) ± standard deviation (SD) = 14.38 ± 4.91; Cronbach’s *α* = 0.82), social (7 items: range 0–21; M ± SD = 18.28 ± 3.43; Cronbach’s *α* = 0.76), emotional (6 items: range 0–18; M ± SD = 14.02 ± 3.66; Cronbach’s *α* = 0.76), and work/life functioning (7 items: range 0–21; M ± SD = 17.30 ± 4.03; Cronbach’s *α* = 0.79). The four domains were summed to create a total score (M ± SD = 64.00 ± 12.85, range 10–81; Cronbach’s *α* = 0.91). Two FACT-G items which assessed the patient’s relationship with her doctor were excluded because this research did not focus on patient-provider interactions. Spiritual wellness was determined by using the first 12 items from the Functional Assessment of Chronic Illness Therapy-Spiritual Wellbeing Scale (FACIT-Sp) [23]; scores ranged from 2 to 36 (M ± SD = 31.34 ± 5.20), and internal consistency was good (Cronbach’s *α* = 0.85) [21]. Response options used for FACIT and FACT-G items were: 0 = not at all to 4 = very much. The recall time frame for these items was the last 7 days.

Level of stress was determined using three items from the 4-item Perceived Stress Scale [24]. This scale was used to measure cancer patients’ perception of their stress during two time windows, the 2–3 months after initial diagnosis and in the month prior to the interview. Depression was measured using five items from the Brief Symptom Inventory [25]. The same 5-point response options were used for the three stress and five depression items. Scores ranged from 0 to 5 (Cronbach *α* = 0.804; M ± SD = 1.67 ± 1.72). Finally, the number of comorbid conditions was the sum of an affirmative response to the following physical conditions self-reported as physician diagnosed: high blood pressure, heart disease, diabetes, irritable bowel syndrome, fibromyalgia, stroke, or liver disease. Only stress and number of comorbid physical conditions at interview were included as outcomes. Stress and depression at diagnosis were excluded as outcome given the potential for biased recall of symptoms experienced on average 9–12 months prior to being interviewed. Stress and depression at diagnosis were investigated as potential confounders (Model 2).

Demographic or cancer-related attributes of participants were provided directly from women during phone interviews and evaluated as potential confounders. These factors included race, education, family income, current and past sexual or partner violence. Additional data was available from the cancer registry (e.g. age at diagnosis, race, stage, cancer site). Stages III–IV were grouped as later stage, while earlier stage was defined to include in situ, for breast cancer only, and I–II.

Information to describe intimate partner violence (IPV) by type (physical, sexual, psychological) and timing (current or past only) was obtained in phone interviews If IPV was disclosed, follow-up questions were asked to determine which partner was abusive (current, partner at cancer diagnosis, or past partner). Items used were based on (a) the revised Conflict Tactic Scale [26] to measure of sexual and physical IPV and (b) modified versions of the Measure of Psychologically Abusive Behaviors (MPAB) [27] and the Women’s Experience with Battering Scale (WEB) [28] as measures of psychological abuse. Adult sexual assaults and childhood sexual abuse were additionally measured (see Table 1 legend for items’ wording) and included with IPV to provide a dichotomous measure of lifetime violence.

**Table 1:** Participant characteristics (*N* = 3320) of women by Appalachian County of residence

Demographic or cancer characteristic	Appalachian (*n* = 990)	Non-Appalachian (*n* = 2330)	*t* test _df_^*p* value*^
Mean age at diagnosis (Standard Error, SE)	57.33 (0.34)	56.46 (0.22)	2.11 _3333_^.04^
Mean days from diagnosis to interview (SE)	389.23 (5.27)	387.68 (3.64)	0.25 _3333_^.80^

	%	%	χ^2^_df_^*p* value*^
North Carolina Resident (vs. Kentucky)	30.8	25.3	10.69 _1_^.001^
Non-white (vs. White)	4.5	11.6	40.38 _1_^< .0001^
Currently married (vs. not married)	63.1	61.9	0.41 _1_^NS^
Late stage at cancer diagnosis (Stage III–IV versus 0, I–II)	24.7	27.1	1.00 _1_^NS^
Current smoker (vs. not a current smoker)	14.8	11.2	9.34 _1_^.002^
Beale code (rurality)			870.51 _4_^< .0001^
Metro or fringe to metro (≥ 1 million)	0.0	49.8	
Metro (< 250,000–1 million)	12.6	25.5	
Urban pop > 20,000	12.5	5.7	
Urban pop < 2000 adjacent to Metro	52.0	15.4	
Rural adjacent to Metro	22.9	3.6	
Education			58.97 _4_^< .0001^
Less than high school graduate	14.5	7.2	
High school graduate-GED	32.4	29.2	
Some college	15.0	18.5	
Vocational school or Associates Degree	13.8	14.0	
College graduate or more	24.3	31.1	
Insurance			56.70 _3_^< .0.0001^
No insurance of any kind	6.3	4.4	
Medicaid versus no Medicaid	13.5	8.1	
Medicare versus no Medicare	31.7	25.6	
Private versus Other or no coverage	48.5	61.9	
Monthly household income			72.01 _5_^< .0.0001^
Less than $1000	15.3	10.4	
$1000–$1999	27.4	20.0	
$2000–$2999	18.9	16.4	
$3000–$3999	12.3	13.4	
$4000–$4999	11.2	15.0	
$5000 or more	15.0	24.8	
IPV timing			
Lifetime violence (any of the forms below)	39.1	41.9	2.69 _1_^NS^
IPV (physical, sexual or psychological)	35.6	37.2	1.88 _1_^NS^
Current IPV	8.3	7.5	0.62 _1_^NS^
Sexual assault^a^	4.9	5.2	0.15 _1_^NS^
Childhood sexual abuse^b^	9.9	11.3	1.39 _1_^NS^
Type of cancer			13.92 _10_^NS^
Leukemia/Lymphoma	3.7	4.9	
Colorectal	10.0	8.3	
Cervical/vulvar	3.4	2.7	
Endometrial	5.6	4.4	
Ovarian	3.2	2.2	
Head/neck/lung	5.6	6.4	
Other GI (stomach, liver, gallbladder, pancreas)	0.7	1.1	
Skin, bone, brain	3.4	3.3	
Bladder/kidney	2.4	2.4	
Thyroid	4.0	4.2	
Breast	57.8	60.1	

*Unadjusted associations. χ^2^ tests used for categorical variables, two-sample *t* test used for continuous variables

^a^Sexual assault item: “Since you turned age 18, did anyone other than an intimate or dating partner ever physically force or otherwise force you to do any sexual activity against your will?”

^b^Childhood sexual abuse was identified based on yes responses to either of the following two items: “Before age 18, (1) did anyone ever physically force, or (2) attempt to physically force you, to do any sexual activity against your will?”

### Statistical analysis

Demographic attributes associated with living in an Appalachian county at diagnosis were assessed using either two-sided *t* tests for continuous variables or Chi square tests for categorical variables (Table 1). These bivariate associations were used to determine potential confounders to be included in multivariable analyses. Because QOL outcome measures were correlated, Multivariate Analysis of Variance (MANOVA) was used. Significant Wilks’ Lambda Statistics indicated that models were appropriate. MANCOVA models were used for the following current health outcome measures: FACT-G total and within domains, FACIT-SP, symptoms of stress and number of comorbid physical conditions. Modification of Appalachian residence by violence was first addressed using MANCOVA model with an interaction term without confounder adjustments (unadjusted model); a *p* value of ≤ .10 for interaction was used. To address the confounding factors associated with Appalachian residence and QOL outcomes, three sets of models were run to sequentially add confounders as follows: Model 1 included demographics or cancer attributes (see Table 2 legend for complete listing), Model 2 additionally included depression and stress at cancer diagnosis, and Model 3 additionally included income, education, and health insurance coverage. Unadjusted outcome means (± SE) by Appalachian residence were presented with associated t test and p values. A significance level of .05 was used for statistical tests; SAS Version 9.4 (Cary, NC) was used for all analyses.

**Table 2:** Appalachian residence and cancer-related quality of life (QOL) within Violence Strata

QOL measures	Unadjusted means (Std Err) by residence		*t* test, ^*p* value^ for difference in means by residence [within Violence Strata]: main effects				
Functional assessment of cancer therapy^A^	Appalachian (*n* = 990)	Non-Appalachian (*n* = 2330)	Interaction: Residence*Violence	Unadjusted	Model 1	Model 2	Model 3
FACT-general total	61.24 (0.42)	63.39 (0.30)	*t* = − 1.19; ^*p*=NS^	*t* = − 4.51, ^p<.0001^	*t* = − 4.33, ^p<.0001^	*t* = − 3.70, ^*p*=.0002^	*t* = − 1.62, ^*p*=.11^
Violence	57.24 (0.64)	59.96 (0.43)		[*t* = − 3.67, ^*p*=.0002^]	[*t* = − 3.63, ^*p*=.0003^]	[*t* = − 2.80, ^*p*=.005^]	[*t* = − 1.49, ^*p*=.14^]
No violence	65.23 (0.52)	66.82 (0.37)		[*t* = − 2.63, ^*p*=.009^]	[*t* = − 2.44, ^*p*=.01^]	[*t* = − 2.45, ^*p*=.01^]	[*t* = − 0.75, ^*p*=NS^]
Physical domain	13.03 (0.16)	13.91 (0.11)	*t* = − 0.29; ^*p*=NS^	*t* = − 4.85, ^*p*<.0001^	*t* = − 4.94, ^*p*<.0001^	*t* = − 4.40, ^*p*<.0001^	*t* = − 2.70, ^*p*=.007^
Violence	12.04 (0.24)	12.98 (0.16)		[*t* = − 3.31, ^*p*=.0009^]	[*t* = − 3.42, ^*p*=.0006^]	[*t* = − 2.67, ^*p*=.008^]	[*t* = − 1.58, ^*p*=.11^]
No violence	14.02 (0.20)	14.85 (0.14)		[*t* = − 3.62, ^*p*=.0003^]	[*t* = − 3.67, ^*p*=.0002^]	[*t* = − 3.74, ^*p*=.0002^]	[*t* = − 2.37, ^*p*=.02^]
Social domain	17.88 (0.11)	18.07 (0.08)	*t* = − 1.43; ^*p*=.15^	*t* = − 1.50, ^*p*=.13^	*t* = − 1.31, ^*p*=.19^	*t* = − 0.73, ^*p*=NS^	*t* = 0.61, ^*p*=NS^
Violence	16.82 (0.17)	17.20 (0.11)		[*t* = − 1.89, ^*p*=.06^]	[*t* = − 1.80, ^*p*=.07^]	[*t* = − 1.20, ^*p*=NS^]	[*t* = − 0.35, ^*p*=NS^]
No violence	18.93 (0.14)	18.94 (0.10)		[*t* = − .06, ^*p*= NS^]	[t = 0.13, ^*p*= NS^]	[t = 0.31, ^*p*=NS^]	[t = 1.40, ^*p*=.16^]
Emotional domain	13.67 (0.12)	14.09 (0.09)	*t* = − 0.39; ^*p*=NS^	*t* = − 3.07, ^*p*=.002^	*t* = − 2.65, ^*p*=.008^	*t* = − 1.77, ^*p*=.08^	*t* = − 0.81, ^*p*=NS^
Violence	12.82 (0.18)	13.30 (0.12)		[*t* = − 2.23, ^*p*=.03^	[*t* = − 2.01, ^*p*=.04^]	[*t* = − 0.96, ^*p*=NS^]	[*t* = − 0.36, ^*p*=NS^]
No violence	14.52 (0.15)	14.89 (0.11)		[*t* = − 2.13, ^*p*=.03^]	[*t* = − 1.74, ^*p*=.08^]	[*t* = − 1.64, ^*p*=.10^]	[*t* = − 0.87, ^*p*=NS^]
Work/life functioning	16.66 (0.14)	17.31 (0.10)	*t* = − 1.80; ^*p*=.07^	*t* = − 4.22, ^*p*<.0001^	*t* = − 4.05, ^*p*<.0001^	*t* = − 3.43, ^*p*=.0006^	*t* = − 1.35, ^*p*=.18^
Violence	15.54 (0.21)	16.48 (0.14)		[*t* = − 3.88, ^*p*=.0001^]	[*t* = − 3.83, ^*p*=.0001^]	[*t* = − 3.12, ^*p*=.002^]	[*t* = − 1.82, ^*p*=.07^]
No violence	17.77 (0.17)	18.15 (0.12)		[*t* = − 1.93, ^*p*=.05^]	[*t* = − 1.74, ^*p*=.08^]	[*t* = − 1.62, ^*p*=.11^]	[t = 0.08, ^*p*=NS^]
FACIT-SP	30.96 (0.17)	31.23 (0.12)	*t* = − 1.33, ^*p*=.18^	*t* = − 1.40, ^*p*=NS^	*t* = − 1.05, ^*p*=NS^	*t* = − 0.27, ^*p*=NS^	t = 0.78, ^*p*=NS^
Violence	29.63 (0.26)	30.16 (0.18)		[*t* = − 1.76, ^*p*=.08^]	[*t* = − 1.58, ^*p*=.11^]	[*t* = − 0.78 ^*p*=NS^]	[*t* = − 0.11, ^*p*=NS^]
No violence	32.29 (0.21)	32.30 (0.15)		[*t* = − 0.06, ^*p*=NS^]	[*t* = 0.28, ^*p*=NS^]	[*t* = 0.54 ^*p*=NS^]	[*t* = 1.40, ^*p*=.16^]
Stress, at interview	3.49 (0.09)	3.22 (0.06)	*t* = 1.64; ^*p*=0.10^	*t* = 2.71, ^*p*=.007^	*t* = 2.72, ^*p*=.007^	*t* = 1.79, ^*p*=.07^	*t* = 0.70, ^*p*=NS^
Violence	4.26 (0.13)	3.83 (0.09)		[*t* = 2.80, ^*p*=0.005^]	[*t* = 2.86, ^*p*=.004^]	[*t* = 1.88, ^*p*=.06^]	[*t* = 1.19, ^*p*=NS^]
No violence	2.71 (0.11)	2.61 (0.08)		[*t* = 0.89, ^*p*=NS^]	[*t* = 0.81, ^*p*=NS^]	[*t* = 0.55, ^*p*=NS^]	[*t* = − 0.34, ^*p*=NS^]
# comorbid conditions	0.99 (0.04)	0.81 (0.03)	*t* = 0.26; ^*p*=NS^	*t* = 4.34, ^*p*<.0001^	*t* = 3.94, ^*p*<.0001^	*t* = 3.68, ^*p*=.0002^	*t* = 1.99, ^*p*=.05^
Violence	1.11 (0.05)	0.93 (0.04)		[*t* = 2.97, ^*p*=.003^]	[*t* = 2.70, ^*p*=.007^]	[*t* = 2.41, ^*p*=.02^]	[*t* = 1.33, ^*p*=.18^]
No violence	0.86 (0.04)	0.70 (0.03)		[*t* = 3.24, ^*p*=.001^]	[*t* = 2.96, ^*p*=.003^]	[*t* = 2.90, ^*p*=.004^]	[*t* = 1.54, ^*p*=.12^]

Outcome A (Appalachia, Model 1): Wilks lambda = 0.989; *F* = 5.31, DF 7,3281, *p* < .0001

Unadjusted Model includes Appalachian residence, violence (lifetime IPV, sexual assault, or childhood sexual abuse; yes vs. no) and interaction term for Appalachian residence and violence, Model 1 additionally adjusts for State registry (dichotomous: North Carolina versus Kentucky), time since cancer diagnosis as design elements, age (continuous: 18–79) and stage at diagnosis (I–IV), cancer site (see Table 1 for sites), race (dichotomous: non-Hispanic White versus other), and current smoking status (dichotomous: current smoker versus not current smoker)

Model 2 additionally adjusts for depression and stress at cancer diagnosis (continuous symptoms scores)

Model 3 additionally adjusts for highest education attainment (ordinal: < high school graduate, high school graduate, some college, college graduate, other including graduate or professional degree), monthly family income (ordinal: < $1000, $1000–$1999, $2000–$2999, $3000–$3999, $4000–$4999, $5000 or more, and private health insurance (yes vs. no)

## Results

Despite the modest differences in how the two state registries provided direct case recruitment, the overall % of cancer cases completing the survey was very similar (KCR: 40.1% [2434 of 6070 reached by phone or mail]; NCCR: 40.9 [901/2203 reached]. Response rates did not differ by Appalachian region within state.

A total of 3333 women participated in the study and 29.9% of the women resided in Appalachian Kentucky or North Carolina. Thirteen women were excluded due to missing data on violence yielding a final analytic sample of 3320.

As anticipated, women who lived in Appalachian counties were more likely to be White, to currently smoke, live in rural counties, have less education, have lower monthly household incomes, and were less likely to have private health insurance than women living in non-Appalachian counties (Table 1). No differences by Appalachian residence were noted by women’s age, time from diagnosis to interview, stage at diagnosis, current marital status, cancer site, nor violence experienced.

While no differences in the frequency of violence were noted by Appalachian residence, violence was investigated as a moderator of the effect of Appalachian residence and QOL outcomes (see Table 2: Appalachia*Violence interaction). As anticipated based on prior findings [21], FACT scores were significantly lower among women experiencing violence across all outcomes (*p* < .0001; not reported in Table 2). The interaction between Appalachia residence and violence bordered on significance (*p* ≤ .10) for the FACT-G work/life functioning and symptoms of stress at interview thus this interaction term was retained for all modeling (Table 2).

Violence was explored as a mediator of the association between Appalachian residence and QOL where MANCOVA models were compared without and with violence. No differences in total FACT-G total scores by Appalachian residence were observed without (*t* test = − 3.95, *p* < .0001) and with adjustment for violence (*t* test = − 4.51, *p* < .0001; not included in tables).

Table 2 provided analytic findings for the investigation of Appalachian residence and QOL outcomes with the identified interaction term (unadjusted model) and across three additional models: inclusion of demographic and cancer attributes (Model 1), additional inclusion of depression and stress at diagnosis (Model 2) and additional inclusion of socioeconomic indicators (Model 3).

Rural residence was not included as a confounder because this factor was synonymous with Appalachia residence in Kentucky. Because the state cancer registries differed somewhat in researchers’ access to subjects, we opted to additionally adjust analyses for registry site. Age, stage at diagnosis, and cancer site were included as confounders, despite their not being associated with Appalachian residence, because these two factors were so strongly associated with QOL outcomes.

In general, unadjusted FACT-G scores were lower (indicating poorer QOL) among Appalachian versus non-Appalachian residents for FACT-G total scores and physical, emotional, and work/life functionality domains (main effects: Table 2). Similarly, higher stress scores and more comorbid physical conditions were reported by participants living in Appalachian versus non-Appalachian counties. The strength of associations between QOL outcomes and Appalachian residence was not diminished when adjusting for non-socioeconomic factors (unadjusted and Model 1 comparisons). With additional adjustment for depression and stress at diagnosis (Model 2), FACT-G total, physical, work/life functionality domains, and comorbid conditions remained associated with Appalachian residence. Finally, when socioeconomic factors were included (Model 3), differences by Appalachian residence remained only for FACT-G physical and number of comorbid physical conditions at interview.

Turning to interactive effects of violence and Appalachian region (Table 2: within violence strata, across models), the pattern described above for Appalachian residence and QOL outcomes was similar within violence strata. Appalachian residence was significantly associated with poorer QOL outcomes (FACT-G total, physical domain, and comorbid conditions) among women experiencing violence and those never experiencing violence (unadjusted, Model 1 and 2). Violence did appear to modify the effect of Appalachian residence on lower work/life functioning and greater stress only among women experiencing violence. After adjusting for socioeconomic indicators, only lower FACT-G physical domain scores remained associated with Appalachian residence and only among women never experiencing violence.

To provide an indicator of the effect size of these associations, semipartial ω^2^ ± 90% CI were calculated for all covariates included in model 3. Symptoms of depression and stress at cancer diagnosis had the highest effect size estimates predicting FACT-G total scores (violence: *ω*^2^ = 0.2064 ± 0.186–0.225) followed by violence (*ω*^2^ = 0.0742 ± 0.061–0.089), and income (*ω*^2^ = 0.044 ± 0.033–0.056). Appalachian residence had a very modest effect size estimate for the FACT-G total score (violence: *ω*^2^ = 0.005 ± 0.002–0.010). Similar findings were observed for other QOL outcomes.

## Discussion

Our findings indicate that socioeconomic differences by Appalachian residence explained regional differences in QOL outcomes with the important exception that poorer physical QOL (physical FACT-G scores and comorbid physical conditions) remained associated with Appalachian residence. While QOL was disproportionately lower among women experiencing violence, violence did not mediate differences in QOL by Appalachian residence. Women experiencing violence and living in Appalachian regions consistently had poorer QOL outcomes yet these differences did not remain after adjusting for socioeconomic factors. In this order, symptoms of depression and stress, violence, and income had the largest effect size on predicting FACT-G scores.

While few studies have explored mental and physical QOL outcomes by Appalachian residence, the report by Burris and Andrykowski [18] was the most comparable to the current study. These authors found that Appalachian Kentucky cancer patients not only had poorer physical health functioning than non-Appalachian state residents but Appalachian residents also displayed poorer mental health functioning and symptoms of anxiety, depression, distress, and emotional problems. While the current study findings concurred with Burris and Andrykowski’s [18] findings that Appalachian residence was associated with poorer physical health, these differences in mental health indicators did not remain in our analyses after adjusting for socioeconomic factors. This observation suggests that while Appalachian residence was highly correlated within income, income and not residence may be the more salient factors explaining differences in QOL.

Prior research investigating QOL by rural versus urban residence can provide relevant comparisons for the current analysis where Appalachian and rural residence were synonymous. When adjusting for socioeconomic factors, Weaver et al. [19] observed that women with breast cancer who lived in rural versus more urban regions had lower FACT-B total and physical subscale scores. Further, rural cancer survivors were significantly more likely to self-report having fair to poor health. In the current analysis without adjusting for socioeconomic factors, Appalachian (rural) residence was associated with lower FACT-G total scores and increased number of comorbid conditions yet when adjusting for socioeconomic factors Appalachian residence was associated only with lower physical FACT-G domain scores. The current study findings were also similar to those of Reid-Arndt and Cox [17] who observed that rural versus urban breast cancer patients experienced lower overall (FACT-B) QOL scores and lower functional wellbeing. Finally, our findings for Appalachian residence were consistent with the only prospective study of depressive symptoms among cancer patients receiving radiation treatment; Schlegel et al. [29] did not find rural residence to be associated with increased depressive symptoms among study subjects followed across three time points.

Limitations in our methodology included defining QOL indicators based on self-reports, which may be biased. However, women are the ultimate authority on their own experiences and perceptions of their own health. QOL measures were obtained only once after diagnosis thus changes over time in QOL measures may be missed. Finally, while response rates were lower than desired (40.6% of those who could be reached by phone or mail), response rates did not differ by state or Appalachian residence. Study staff did not have access from cancer registries to cases’ cell phone numbers or email addresses. Having these updated contact methods could have increased contact rates.

Confounding bias was unlikely to explain these findings, given measurement and extensive adjustment for covariates. Sampling from population-based cancer registries improved study power and sample representativeness. This study represented the largest to date to explore differences in QOL indicators by Appalachian residence, within strata of violence, and to adjust for socioeconomic factors which so strongly distinguish Appalachian versus non-Appalachian residence.

Southern and rural Appalachia has been characterized by close kinship-based connections, strong religious affiliations, and informal community networks [30]. Our finding no differences in FACIT-SP or FACT-G social domain may be explained by strong religious or social connections available for many in Appalachia. Recent studies among adults in living in Appalachian or other rural settings suggested that close social networks had both positive as well as negative influences on health [31, 32] or seeking mental health services [33].

From the current research, socioeconomic factors appear to mediate Appalachian disparities in poorer cancer QOL. Poverty and physical isolation are realities for many living in Southern and rural Appalachia, and many Appalachians are quite resilient [34]. While rates of violence were not higher in Appalachian regions, Appalachian residence and violence experienced appeared to have a magnifying effect on selected QOL outcomes.

In this context what do our findings mean for cancer survivors and their clinical care providers? Logistic barriers to receipt of recommended cancer treatment for those living in poverty, in rural areas, or experiencing violence include travel distances and costs, limited medical insurance coverage, and other financial barriers. Stigma associated with help-seeking particularly for those in rural areas characterized as “keeping one’s problems to oneself” may be another barrier to receipt of recommended cancer care [35] or psychosocial services. The clinical implications include the need for cancer care teams to consider cancer patients’ residence, socioeconomic resources, symptoms of depression or stress at diagnosis and violence experienced when working with patients and families to strategize cancer treatment plans. Asking about lifetime IPV and SV and providing confidential referrals to community resources may be another important intervention to address the ongoing violence or mitigate continuing health impacts of past violence. Now required distress screening for oncology patients may be a seamless entre to both depression and stress assessment at diagnosis and violence screening. Distance to care, insurance coverage, and having sufficient income to take time to receive care are all important factors in receipt of recommended cancer care. Cancer patients need both appropriate (a) medical care and (b) support services, regardless of their income, residence, or violence experienced. Efforts to ensure access to cancer care through safe-guarding public and affordable private medical insurance are essential to address poverty directly as the source of disparities in cancer outcomes in Appalachia and among rural or other minority populations. Patient navigation and other psycho-oncology support services during the pre and post-treatment transition phase have been shown to significantly improve the quality of life for cancer patients and their families [36]. Increasing access to patient navigation and other psycho-oncology support services in medically underserved and rural populations via electronic or other tele-medical communications could ensure the promise of improving QOL for all cancer patients.
